# Evaluation of the Double-Tracer Gas Single-Breath Washout Test in a Pediatric Field Study

**DOI:** 10.1016/j.chest.2023.09.006

**Published:** 2023-09-15

**Authors:** Anne-Christianne Kentgens, Johanna M. Kurz, Rebeca Mozun, Jakob Usemann, Eva S.L. Pedersen, Claudia E. Kuehni, Philipp Latzin, Alexander Moeller, Florian Singer, The LuftiBus In the School (LUIS) Study Group, Alexander Moeller, Jakob Usemann, Philipp Latzin, Florian Singer, Johanna Kurz, Claudia E. Kuehni, Rebeca Mozun, Cristina Ardura-Garcia, Myrofora Goutaki, Eva S.L. Pedersen, Maria Christina Mallet, Kees de Hoogh

**Affiliations:** a Division of Respiratory Medicine and Allergology, Department of Pediatrics, Inselspital, Bern University Hospital, University of Bern, Bern, Switzerland; b Graduate School for Health Sciences, University of Bern, Bern, Switzerland; c Institute of Social and Preventive Medicine, University of Bern, Bern, Switzerland; d Department of Intensive Care and Neonatology and Children’s Research Center, University Children`s Hospital Zurich, University of Zurich, Zurich, Switzerland; e Department of Respiratory Medicine, University Children`s Hospital Zurich, University of Zurich, Zurich, Switzerland; f University Children’s Hospital Basel (UKBB), Basel, Switzerland; g Division of Pediatric Pulmonology and Allergology, Department of Pediatrics and Adolescent Medicine, Medical University of Graz, Graz, Austria

**Keywords:** adolescent, child, helium, lung function tests, small airway remodeling, sulfur hexafluoride, ventilation tests, wheezing

## Abstract

**Background:**

The early life origins of chronic pulmonary diseases are thought to arise in peripheral small airways. Predictors of ventilation inhomogeneity, a proxy of peripheral airway function, are understudied in schoolchildren.

**Research Question:**

Is the double-tracer gas single-breath washout (DTG-SBW) measurement feasible in a pediatric field study setting? What are the predictors of the DTG-SBW-derived ventilation inhomogeneity estimate in unselected schoolchildren?

**Study Design and Methods:**

In this prospective cross-sectional field study, a mobile lung function testing unit visited participating schools in Switzerland. We applied DTG-SBW, fraction of exhaled nitric oxide (Feno), and spirometry measurements. The DTG-SBW is based on tidal inhalation of helium and sulfur-hexafluoride, and the phase III slope (SIII_He-SF6_) is derived. We assessed feasibility, repeatability, and associations of SIII_He-SF6_ with the potential predictors of anthropometrics, presence of wheeze (ie, parental report of one or more episode of wheeze in the prior year), Feno, FEV_1_, and FEV_1_/FVC.

**Results:**

In 1,782 children, 5,223 DTG-SBW trials were obtained. The DTG-SBW was acceptable in 1,449 children (81.3%); the coefficient of variation was 39.8%. SIII_He-SF6_ was independently but weakly positively associated with age and BMI. In 276 children (21.2%), wheeze was reported. SIII_He-SF6_ was higher by 0.049 g.mol.L^−1^ in children with wheeze compared with those without and remained associated with wheeze after adjusting for age and BMI in a multivariable linear regression model. SIII_He-SF6_ was not associated with Feno, FEV_1_, and FEV_1_/FVC.

**Interpretation:**

The DTG-SBW is feasible in a pediatric field study setting. On the population level, age, body composition, and wheeze are independent predictors of peripheral airway function in unselected schoolchildren. The variation of the DTG-SBW possibly constrains its current applicability on the individual level.

**Trial Registration:**

ClinicalTrials.gov; No.: NCT03659838; URL: www.clinicaltrials.gov

## Graphical Abstract

**Figure:**
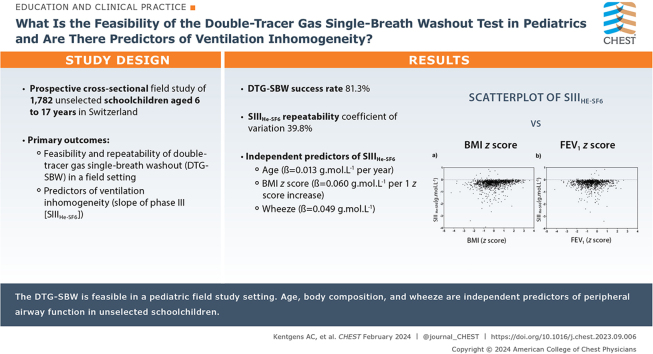

FOR EDITORIAL COMMENT, SEE PAGE 241

**Take-home Points**

**Study Question:** In a large pediatric field study of unselected schoolchildren, what are the success rates and test variation of the double-tracer gas single-breath washout (DTG-SBW) measurement and what are the predictors of ventilation inhomogeneity estimated by the DTG-SBW?

**Results:** We found an acceptable success rate; substantial test variation; and identified age, body composition, and wheeze as independent but relatively weak predictors of ventilation inhomogeneity.

**Interpretation:** The test variation currently constrains the use of the DTG-SBW in children. However, the current data suggest that schoolchildren with wheeze have alterations in ventilation inhomogeneity which can be attributed to peripheral airway dysfunction.

The early life origins of respiratory diseases (eg, COPD) are thought to arise in small airways of lung periphery.^1^ Because of practical constrains, predictors of peripheral airway function (ie, ventilation inhomogeneity) remain understudied in large pediatric populations. The double-tracer gas single-breath washout (DTG-SBW) test may overcome these constraints. The DTG-SBW is a simple lung function test based on tidal inhalation and exhalation of Helium (He) and sulfur-hexafluoride (SF_6_).^2^^,^^3^ The derived slope of phase III (SIII_He-SF6_) measures ventilation inhomogeneity of He and SF_6_, which differ in diffusive gas mixing properties in small airway compartments.^2^^,^^3^ The SIII_He-SF6_ measurement is reliable in research settings and captures altered ventilation inhomogeneity in children with asthma or cystic fibrosis.^2^, ^3^, ^4^, ^5^, ^6^

DTG-SBW may be a simple and accessible tool to allow for early detection of lung function alterations (ie, ventilation inhomogeneity) associated with negative respiratory disease outcomes. However, in unselected pediatric populations, feasibility and repeatability of the DTG-SBW, and predictors of the SIII_He-SF6_, are unknown. Possible predictors of ventilation inhomogeneity constitute age, sex, body composition, wheeze, airflow limitation, and airway inflammation.^7^, ^8^, ^9^ Previous studies suggest that high BMI is associated with dysanaptic lung growth, a nonproportional growth of the airways and lung, because adipose tissue and proinflammatory mediators affect lung growth and development. Pediatric wheeze and airflow limitation increase the risk of COPD in adults.^10^

This study addressed the following two research questions: (1) Is the DTG-SBW measurement feasible in a pediatric field study setting?, and (2) What are the predictors of the DTG-SBW-derived ventilation inhomogeneity estimate in a sample of schoolchildren? To accomplish this, we applied the DTG-SBW test in a large pediatric field study to assess its feasibility and reliability, and explore associations between SIII_He-SF6_ and anthropometric variables, wheeze, and standard lung function indexes.

Previous estimates of feasibility and intratest variability of the nitrogen single-breath washout test in children and adults ranged from 74% to 89% and 13% to 24%, respectively.^11^^,^^12^ For multiple-breath washout, the success rates ranged between 50% and 100% in children.^13^, ^14^, ^15^ We hypothesized that the feasibility and intratest variability of the DTG-SBW applied in unselected schoolchildren in a field study setting were > 75% and < 25%, respectively.

We further hypothesized that SIII_He-SF6_ is associated with age and body composition,^7^ wheeze,^9^^,^^16^ spirometry indexes, and fraction of exhaled nitric oxide (Feno).

## Study Design and Methods

LuftiBus in the School (LUIS) is a prospective cross-sectional observational field study in unselected school-aged children (ClinicalTrials.gov No. NCT03659838).^17^ Inclusion criteria were 6 to 17 years of age, German language skills, and consent to participate. There were no predefined exclusion criteria. A mobile lung function testing unit (motorbus) visited 37 schools in the canton of Zurich, the most populated canton in Switzerland, between 2013 and 2016.^17^ Most children were born in Switzerland (88%) and predominantly of White European ancestry (75.8%). The distribution of the Swiss socioeconomic position index (Swiss-SEP) for families participating in the study was representative to the Swiss-SEP distribution from families with at least one child living in the household from the canton of Zurich.^17^ LUIS took place throughout different seasons (e-Fig 1). A consecutively recruited convenience sample of the whole population was studied because the hardware for DTG-SBW including tracer gas supply became available later during the study. Details about study design, sample size estimates, and data collection have been previously described.^17^ Children performed lung function tests in the following sequence: DTG-SBW, Feno measurement, and spirometry. The ethics committee of the canton of Zurich approved the study (KEK-ZH-Nr No. 2014-0491). Parents or caregivers signed the informed consent form. Children assented verbally and those aged ≥ 15 years also signed the informed consent form.

Anthropometrics were measured on the bus on-site, and parental questionnaires were used to collect information on exposures, respiratory symptoms, diagnoses, and prescribed medication.^17^ Wheeze was specified as parental report of continuous whistling sound during expiration during one or more episodes in the past 12 months.^17^

Tidal DTG-SBW was performed in triplet using the Exhalyzer D (EcoMedics AG) according to recommendations.^18^ An inert double-tracer gas mixture containing 5% SF_6_, 26.3% He, 21% oxygen, and balance nitrogen was inhaled during a single tidal breath and tidally exhaled to functional residual capacity. The setup, protocol, and quality control criteria were in accordance with the European Respiratory Society consensus on inert gas washout testing and were previously described.^3^^,^^17^^,^^18^ The DTG-SBW was analyzed automatically followed by quality control using a customized software platform (LungSim based on Matlab R2014a [The MathWorks Inc]).^17^ Quality control was performed by two trained lung function technicians and included central overread*.* The DTG-SBW trials were categorized according to the quality control categories of A, B, or failed. The quality control protocol used can be found in e-Table 1. Only children who achieved at least two acceptable DTG-SBW trials were included.

The primary outcome measure was the mean SIII_He-SF6_ of all technically acceptable DTG-SBW curves of each subject. SIII_He-SF6_ was computed from the volumetric expirogram by fitting a linear regression slope to the molar mass signal between 65% and 95% of the expired volume. In addition, SIII_He-SF6_ was normalized for expired volume by multiplication with the expired tidal volume as a secondary outcome.^17^ Findings are reported in e-Appendix 1. Both lower and higher SIII_He-SF6_ values compared with a healthy reference population have been shown to be associated with ventilation inhomogeneity arising in central or peripheral airways, respectively.^2^, ^3^, ^4^, ^5^, ^6^

Feno (parts per billion [ppb]) was measured according to recommendations using a single-breath online method and a fast response chemiluminescence analyzer (CLD 88; EcoMedics AG).^19^ Further details on test performance and quality control have been previously described.^17^ Feno is a proxy of eosinophilic airway inflammation; Feno values ≥ 20 ppb can be considered elevated.^20^

Spirometry was performed using a standard spirometer (Masterlab; Jaeger) according to recommendations.^21^ Indices were FEV_1_ and FEV_1_/FVC. Values were expressed as *z* score according to Global Lung Function Initiative reference equations.^17^^,^^22^ Lower limit of normal of FEV_1_ and FEV_1_/FVC were set at ≤ −1.645 *z* score as recommended.^21^^,^^22^

### Analysis

Discrete variables were expressed as count (percentage), and continuous variables were expressed as mean ± SD or median (interquartile range [IQR]), as appropriate. Missing data were not imputed.^17^ Between-group differences were assessed using unpaired *t* tests for parametric estimates and Wilcoxon-Mann-Whitney test for nonparametric estimates. DTG-SBW test feasibility was determined as the success rate calculated as the percentage of children with at least two acceptable trials of all children attempting the test. Intratest repeatability was calculated as coefficient of variation. The success rate of DTG-SBW was calculated as the number of successful DTG-SBW trials as a percentage of all DTG-SBW trials performed per subject.

Associations were assessed using scatterplots, Pearson correlations, and univariable linear regression models. Potential predictors of SIII_He-SF6_ included age, sex, height, weight, and BMI *z* score; wheeze; and Feno, FEV_1_, and FEV_1_/FVC. A multivariable linear regression model was used to explore these variables as independent predictors of SIII_He-SF6_. Variables were analyzed as continuous variables with their original scale, wheeze as a binary variable (ie, yes or no), and Feno as quintiles ensuring balanced observations per category. Regression model diagnostics were used to confirm underlying assumptions. *P* < .05 was considered statistically significant. All analyses were performed using STATA (USA Version 16.0; StataCorp LP). Figures were made using GraphPad Prism version 8.0.1 (GraphPad Software).

## Results

**Figure 1:**
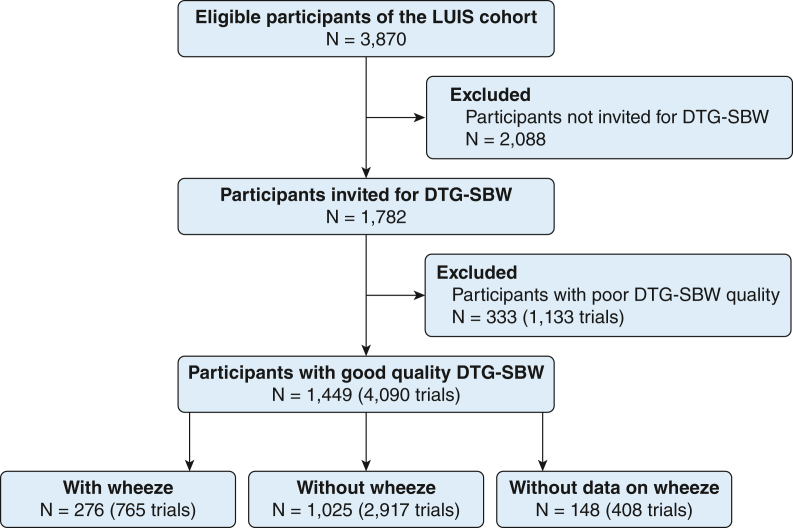
Flowchart of study participants and success rate of DTG-SBW. Out of the 3,870 children of the LUIS study, 1,782 children performed DTG-SBW (46%). Of these children, 1,449 children had acceptable DTG-SBW data (81%). DTG-SBW = double-tracer gas single-breath washout; Feno = fraction of exhaled nitric oxide; LUIS = LuftiBus in the School.

In total, 3,870 children were enrolled into the LUIS study (Fig 1). The children’s median age was 12.1 years (IQR, 9.3-14.0 years), and one-half of the population were female. The DTG-SBW test was applied in 1,782 children (46.0%), who were slightly younger (0.7 years), had slightly lower Swiss-SEP (1.3 points), reported hay fever somewhat more frequently (2.7%), and had slightly lower Feno (2.6 ppb) than children not invited to perform the DTG-SBW. There were no systematic differences in anthropometric and lung function estimates between these children (e-Table 2). Anthropometric characteristics and lung function estimates can be found in Table 1 and e-Table 2.

**Table 1:** Characteristics of Study Participants

Variable	LUIS Study (N = 3,870)	Invited for DTG-SBW (n = 1,782)	Acceptable DTG-SBW Data (n = 1,449)
General characteristics			
Male sex	1,937 (50.1)	889 (49.9)	719 (49.6)
Age, y	12.1 ± 2.7	11.7 ± 2.8	11.9 ± 2.7
BMI, *z* score	0.1 ± 1.2	0.1 ± 1.1	0.1 ± 1.1
White ethnicity	2,933 (75.8)	1,349 (75.7)	1,107 (76.4)
Swiss-SEP	69.5 (62.1-75.9)	69.5 (62.1-75.9)	69.4 (62.1-75.0)
Wheeze	735 (19.0)	322 (18.1)	276 (19.1)
Hay fever	767 (19.8)	326 (18.3)	277 (19.1)
Atopic dermatitis	401 (10.4)	188 (10.6)	160 (11.0)
Asthma diagnosis	293 (7.6)	135 (7.6)	115 (7.9)
Asthma medication	577 (14.9)	262 (14.7)	218 (15.0)
Lung function			
Feno, ppb	12.3 (7.2–21.5)	11.0 (6.3-19.6)	11.1 (6.1-19.7)
FEV_1_, *z* score	−0.5 ± 1.0	−0.52 ± 0.97	−0.54 ± 0.97
FEV_1_/FVC, *z* score	−0.2 ± 1.1	−0.25 ± 1.04	−0.24 ± 1.06
SIII_He-SF6_, g.mol.L^−1^		−0.30 ± 0.54	−0.30 ± 0.42

Data are presented as mean ± SD, No. (%), or median (interquartile range). All questionnaire data were parent reported. Asthma medication included any inhaled corticosteroids or short-acting or long-acting beta-agonists or systemic treatment (eg, leukotriene receptor antagonists). DTG-SBW = double-tracer gas single-breath washout; Feno = fraction of exhaled nitric oxide; LUIS = LuftiBus in the School; ppb = parts per billion; SIII_He-SF6_ = phase III slope; Swiss-SEP = Swiss socioeconomic position index.

### Feasibility and Repeatability

In total, 5,223 DTG-SBW trials were obtained, of which 4,090 trials (78.3%) were of acceptable quality. Therefore, 1,449 out of 1,782 children (81.3%) successfully achieved DTG-SBW tests (e-Tables 3-5). DTG-SBW success rate was higher than the hypothesized success rate (75%). Children with successful DTG-SBW tests were 1.1 years older, had a lower Swiss-SEP, and reported wheeze more often than the children with unsuccessful tests; all other anthropometric and questionnaire data were comparable (e-Table 4).

In children with a successful DTG-SBW test, trial quality was rated higher more often. Frequency of higher trial quality control categories was associated with the number of acceptable trials (e-Fig 2, e-Tables 6-8) until a maximum of four trials. The mean SIII_He-SF6_ ± SD was −0.30 ± 0.42 g.mol.L^−1^. The repeatability of SIII_He-SF6_ with a median intratest coefficient of variation of 39.8% (IQR, 22.0%-70.9%) was poorer than the hypothesized repeatability (25%). For more details on DTG-SBW feasibility and repeatability, we refer to e-Figure 3 and e-Table 9.

### Predictors of Ventilation Inhomogeneity

**Figure 2:**
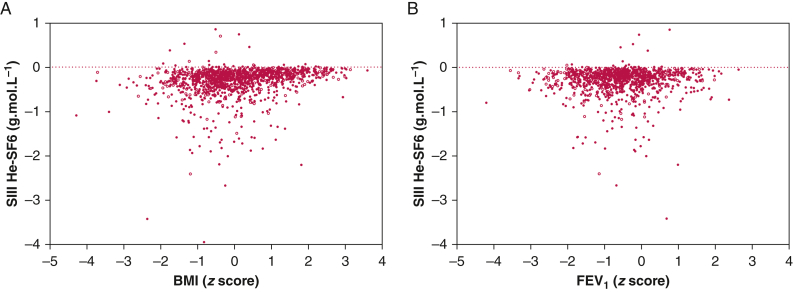
A, B, Scatterplot of the double-tracer gas single-breath washout-derived SIII_He-SF6_ vs BMI (A) and FEV_1_ (B). BMI and FEV_1_ are expressed as *z* score. The closed circles display SIII_He-SF6_ values of children without wheeze, and open circles display values of children with wheeze. We have excluded one outlier (BMI = −1.7 *z* score and SIII_He-SF6_ = 2.9 g.mol.L^−1^) in panel A to ease visualization. SIII_He-SF6_ = phase III slope.

SIII_He-SF6_ was associated with all preselected anthropometric variables except for sex. In univariable regression models, SIII_He-SF6_ was positively associated with age, height, weight, and BMI *z* score (Fig 2, Table 2). In a multivariable regression model, only age and BMI remained independent predictors of SIII_He-SF6_, increasing SIII_He-SF6_ by 0.013 g.mol.L^−1^ per 1-year increase in age and by 0.060 g.mol.L^−1^ per 1 *z* score increase in BMI, respectively.

**Table 2:** Nonadjusted and Adjusted Association Between SIII_He-SF6_ and Potential Predictors

Predictor	Regression Coefficient	95% CI	*P* Value^a^
Anthropometrics			
Sex, male vs female	−0.011	−0.050 to 0.028	.592
Age, y	0.017	0.010 to 0.024	< .001^a^
Height, cm	0.004	0.003 to 0.005	< .001^a^
Weight, kg	0.005	0.004 to 0.006	< .001^a^
BMI, *z* score	0.067	0.053 to 0.086	< .001^a^
Symptoms			
Wheeze vs no wheeze	0.072	0.024 to 0.120	.003^a^
Wheeze vs no wheeze, adjusted	0.049	0.002 to 0.096	.042
Lung function			
Feno, quintiles	0.004	−0.010 to 0.018	.557
FEV_1_, *z* score	0.012	−0.008 to 0.032	.255
FEV_1_/FVC, *z* score	0.005	−0.013 to 0.023	.606

Associations between SIII_He-SF6_ and potential predictors were assessed using univariable and multivariable linear regression models. Predictors were age, sex, height, weight, and BMI and wheeze, Feno, FEV_1_, and FEV_1_/FVC. Wheeze was included as a binary variable (ie, yes or no), and Feno was included as data-driven quintiles ensuring balanced observations per category; all other variables were included as continuous variables with their original scale. The quintile boundaries for Feno were as follows: 0.0 to 4.9, 5.0 to 8.8, 8.9 to 13.8, 13.9 to 23.4, and 23.5 to 197.0 parts per billion, respectively. A multivariable linear regression model was used to assess which anthropometric variables were independent predictors of SIII_He-SF6_, and the independent predictors age and BMI were used to adjust the association of SIII_He-SF6_ with wheeze. All associations described the change in SIII_He-SF6_ in g.mol.L^−1^ induced by 1-unit increase in the predictor. Feno = fraction of exhaled nitric oxide; SIII_He-SF6_ = phase III slope.

a Statistically significant difference (< .05).

In total, 276 children reported wheeze, 1,025 children had no wheeze, and 148 children had missing information regarding wheeze and were excluded from this analysis (Fig 1). Children with wheeze were slightly older (0.7 years), heavier (BMI, 0.2 *z* score), and reported atopic diseases more frequently (e-Table 10).

Feno was slightly higher (4.3 ppb) and spirometry was lower (FEV_1_, 0.21 *z* score) in children with wheeze than in children without wheeze (e-Table 10). SIII_He-SF6_ was associated with wheeze in univariable regression models, and it remained weakly positively associated with wheeze after adjustment for age and BMI *z* score (Table 2). SIII_He-SF6_ was higher by 0.049 g.mol.L^−1^ in children with wheeze compared with those without, but it was not associated with Feno or with the spirometry indices FEV_1_ and FEV_1_/FVC (e-Table 11, Table 2). A post hoc analysis in a subgroup of children with a BMI *z* score > 1.0 showed similar results compared with the primary analysis in the whole cohort (e-Table 12).

## Discussion

In this large pediatric field study setting, we found that the DTG-SBW measurement was feasible in a mobile bus lung function laboratory. Repeatability was poorer than hypothesized. We identified predictors of ventilation inhomogeneity in unselected schoolchildren. SIII_He-SF6_ was weakly positively associated with age, BMI, and wheeze but not with Feno or spirometry indices. On the population level in sufficiently large samples such as in our study, SIII_He-SF6_ captures a subtle signal of alterations in ventilation inhomogeneity, suggesting small airways dysfunction in children with wheeze. However, on the individual level, SIII_He-SF6_ does not seem sensitive enough to screen for alterations in ventilation inhomogeneity in unselected children.

### Interpretation

In this field study, we found an acceptable success rate in unselected schoolchildren. The current success rate was higher than hypothesized (75%) but lower than previously reported (92%) in selected children within research laboratory settings.^2^ Because of the field study conditions with possibly a more distracting environment than standard laboratories and children naive to the use of sealed mouthpieces, success rates were somewhat lower. This is supported by the observed learning effect during testing in this study. Previously reported success rates of other tidal breathing protocols were similar to our findings.^23^ In our study, the reason for DTG-SBW test failure was mainly variable breathing pattern. Because of time constraints, details of test failure were not recorded on-site. In a previous study performed in a lung function laboratory, variable breathing pattern accounted for 94% of DTG-SBW test failures in school-aged children.^2^ In that study, reasons for DTG-SBW test rejection were (1) variable tidal flows and volumes, (2) small tidal volumes lacking phase III of the expirogram, and (3) technical errors.^2^

The coefficient of variation quantifying intratest variability of SIII_He-SF6_ was higher than previously reported (19%) for DTG-SBW,^2^ but comparable with the phase III slope indices Scond and Sacin from the established multiple-breath washout test, supporting the reliability of the current analysis.^6^^,^^24^ The estimated mean value of SIII_He-SF6_ was close to zero in our study; therefore, small changes may have increased the coefficient of variation exponentially. The variability seen can be because of factors related to the field study setting, but estimation of the proportion of variability that can be attributed to the setting is challenging. It is well established, however, that the intratest variability for inert gas analysis is high, commonly thought to be because of effects of breathing. Interestingly, variability of SIII_He-SF6_ was associated with age and the variability in tidal volume in our study, but not with other potential explanatory variables (eg, the SIII_He-SF6_ value itself). These data suggest that phase III slope indices are prone to considerable inherent physiologic variability and tidal breathing. Normalization for tidal volume alone may not substantially decrease variability or increase sensitivity of the test.^22^^,^^25^^,^^26^ Current protocols for phase III slope measurement seem to require refinement prior to clinical routine application. The high intratest variability may dampen test sensitivity to estimate subtle physiologic signals in individuals. Further research is needed to identify potentially modifiable sources of test variability and assess the potential of alternate protocols to reduce intratest variability of the DTG-SBW.

Additionally, previous data demonstrated that SIII_He-SF6_ correlates with standard estimates of ventilation inhomogeneity.^2^, ^3^, ^4^, ^5^, ^6^^,^^24^ However, it is unclear whether SIII_He-SF6_ is also a proxy of structural airway disease. Although it is established that in cystic fibrosis the lung clearance index correlates with structural airway changes detected in chest CT scan, there is one negative study for SIII_He-SF6_.^27^ Multiple-breath washout or lung imaging were not obtained in this field study. However, these estimates would have allowed more in-depth assessment of the diagnostic performance of SIII_He-SF6_. Our study provides further evidence that body composition is a predictor of lung function development. Our data are in line with previous findings suggesting age-dependent or height-dependent effects on ventilation inhomogeneity estimates (eg, lung clearance index from multiple-breath washout).^7^^,^^28^ Our data further suggest that unfavorable body composition estimated by BMI may modify ventilation inhomogeneity. Reasons remain speculative but may partly relate to airway dysanapsis observed in children with high BMI.^28^ Indeed, we have recently shown that the spirometry indexes obtained in this cohort did not fit well the reference values from the Global Lung Function Initiative.^26^ Underestimation of FEV_1_ and FVC in the current cohort was partly explained by BMI; however, FEV_1_/FVC was not affected.

Wheezy symptoms are common and account for considerable burden in pediatric health care. We found altered ventilation inhomogeneity possibly arising in obstructed small airways related to previous wheezy symptoms.^2^^,^^4^, ^5^, ^6^ Interestingly, our study suggests that these alterations in ventilation inhomogeneity were independent of airway inflammation or airflow limitation. However, overlap in SIII_He-SF6_ values of children with vs without wheeze was considerable. Comparable with other studies, peripheral airway function estimated by current inert gas tests appears largely normal in children with wheeze.^29^ Therefore, the difference in SIII_He-SF6_ in children with wheeze was relatively small, and adjustment for age and BMI further increased the CIs. Comparable with SIII_He-SF6_, Feno, FEV_1_, and FEV_1_/FVC values were overlapping between children with vs without wheeze, suggesting overall relatively low pretest probability (ie, low prevalence) of lung function abnormalities in the current cohort.

### Strengths and Limitations

The large sample size is a strength of this prospective study because it allows conclusive analyses of potential predictors of lung function. Our study allowed for thorough assessment of potential predictors of the SIII_He-SF6_ estimate, including anthropometric and lung function measures. The large sample of unselected schoolchildren supports the generalizability of our findings. Participation of schools was decided by the heads of the schools, which may have introduced selection to some extent. However, the Swiss-SEP for families participating in the study was representative for the canton of Zurich.^11^ Because the DTG-SBW test was introduced later in this study, only a subgroup of the LUIS study was invited to perform DTG-SBW. During this study period, the frequency of measurements varied over time. SIII_He-SF6_ was not influenced by timing of measurements (ie, seasonal effects).

The current protocol determined the sequence of testing to avoid influences from forced breathing maneuvers during spirometry on SIII_He-SF6_ and Feno. Tidal inhalation of inert gas during the DTG-SBW unlikely influenced subsequent Feno or spirometry measurements.

We report wheeze in 19% of our study population, whereas this was 8% for the total LUIS population. In the latter study, wheeze was defined as whistling or panting sound originating from the chest within the last 12 months. In the current analysis, we expanded the definition of wheeze by adding whistling or panting sound originating from the chest in response to triggers (eg, exercise, respiratory tract infection, cold air, other).

The proportion of variation in SIII_He-SF6_ in this unselected population that can be explained by wheeze was low. We acknowledge that questionnaire-based classification of wheeze may have been subject to recall and misclassification bias. Parent-reported wheeze may have been less precise than physician-reported wheeze. The sound of wheezing that parents notice unaided by a stethoscope (ie, audible wheeze) originates from trachea and larger bronchi, rather than from the peripheral small airways. We assume that misclassification rather led to underestimation of the strength of association between wheeze and SIII_He-SF6_. Premature birth may affect lung development and alter ventilation inhomogeneity in some children. We were unable to explore possible effects of prematurity on SIII_He-SF6_.

## Interpretation

Our results suggest that DTG-SBW is feasible in children between 6 and 17 years of age. Data from younger children are scant and warrant further study.^2^^,^^4^^,^^30^ Despite good feasibility, the high variability and presumably low sensitivity to capture slightly increased ventilation inhomogeneity constrain its use in unselected individuals. Currently, the DTG-SBW is applicable in research settings and sufficiently large populations, or in selected individuals with high pretest probability of lung function abnormalities. In the latter, we have shown that SIII_He-SF6_ is responsive to bronchodilator inhalation in asthma or chest physiotherapy in cystic fibrosis.^2^, ^3^, ^4^, ^5^, ^6^ Distinct interpretation of dynamics in SIII_He-SF6_ warrants further research. Future longitudinal studies are warranted to establish the minimal clinically important differences derived from variability estimates and patient-reported outcomes.

To conclude, the DTG-SBW measurement is feasible in pediatric field studies. However, relatively high variability of SIII_He-SF6_ appears to limit the interpretation. This makes DTG-SBW currently unsuitable in small populations with low pretest probability of impaired lung function. In the current relatively large population of unselected schoolchildren, age, body composition, and wheeze were identified as predictors of ventilation inhomogeneity estimated by SIII_He-SF6_. Schoolchildren with wheeze may have alterations in ventilation inhomogeneity which can be attributed to peripheral airway dysfunction.

## Funding/Support

Study setup, development, and data collection were funded by Lunge Zürich, and the analysis was funded by grants from Lungenliga Bern and Foundation KinderInsel. A.-C. K. is recipient of a Swiss Excellence Grant from the Swiss government.

## Financial/Nonfinancial Disclosures

The authors have reported to *CHEST* the following: A.-C. K. is recipient of a Swiss Excellence Grant from the Swiss government. J. M. K. reports funding for this work from a grant from the KinderInsel Bern Foundation. J. U. reports receiving grants or contracts from the Swiss Lung Foundation, Palatin Foundation, University of Basel, and Swiss Cancer League; and payment or honoraria for lectures, presentations, speaker bureaus, manuscript writing, or educational events received from Vertex and the Zürich Lung Foundation, outside the submitted work. P. L. reports receiving grants or contracts from Vertex and OM Pharma paid to the institution; personal payment or honoraria and payments or honoraria for lectures, presentations, speaker bureaus, manuscript writing, or educational events received from Vertex, Vifor, and OM Pharma; personal fees and fees paid to the institution for participation on a data safety monitoring or advisory board for Polyphor, Vertex, OM Pharma, and Vifor; and personal fees for participation on data safety monitoring or advisory board for Santhera (DMC) and Sanofi Aventis. A. M. reports receiving consulting fees from Vertex Pharmaceuticals and Vifor Pharma; payments or honoraria for lectures, presentations, speaker bureaus, manuscript writing, or educational events received from Vertex Pharmaceuticals and Vifor Pharma; participation on a data safety monitoring or advisory board for Vertex Pharmaceuticals; leadership or fiduciary roles in other boards, societies, committees, or advocacy groups, paid or unpaid, held for European Respiratory Society Assembly 7, Swiss Society of Pulmonology Board, Swiss Society of Pediatric Pulmonology Board, Swiss Working Group for Cystic Fibrosis, and Swiss Society for Sleep Research, Sleep Medicine and Chronobiology; and receipt of medical writing from Vertex Pharmaceuticals, with all disclosures made outside the submitted work. F. S. reports support of this manuscript from the Medical University of Graz for the processing charges; grants or contracts from the Medical University of Graz and Lungen Liga Bern paid to the institution; personal payment or honoraria for lectures, presentations, speakers bureaus, manuscript writing, or educational events from Novartis Pharma Switzerland, Vertex Pharmaceuticals Switzerland, and Vertex Pharmaceuticals Austria; and nonfinancial support from EcoMedics AG, Dürnten, Switzerland and Chiesi Pharmaceuticals Austria, outside the submitted work. None declared (R. M., E. S. L. P., C. E. K.).

## Supplementary Data

e-Online Data
