# Supplementary material for: Evaluation of the Double-Tracer Gas Single-Breath Washout Test in a Pediatric Field Study
Source: Chest. 2023 Sep 15;165(2):396–404. doi: 10.1016/j.chest.2023.09.006 (PMC10851274; doi:10.1016/j.chest.2023.09.006)
Supplement: e-Online Data [file mmc1.docx]

**ONLINE SUPPLEMENT**

**Evaluation of the double tracer gas single-breath washout test in a pediatric field study**

**Abbreviations**

BMI body mass index

DTG-SBW double-tracer gas single-breath washout

FEV_1_ forced expiratory volume in the first second

FVC forced vital capacity

FeNO fraction of exhaled nitric oxide

He helium

IQR interquartile ranges

ppb parts per billion

SIII phase III slope

SnIII phase III slope normalized for expired tidal volume

SD standard deviations

SF_6_ sulfur-hexafluoride

**DTG-SBW success rate**

In total, 1449 (81.3%) out of 1782 children successfully achieved double tracer gas single-breath washout (DTG-SBW) tests consisting of at least two acceptable trials scored A or B as per quality control rating (e-Tables 1-3). In the current study, 333 children excluded from the analysis achieved (mean) 2.5 trials of which 26.9% were of acceptable quality. 190 (57.1%) out of these 333 children achieved one of at least two acceptable trials. For the included children, 3.0 trials were achieved on average, with 93.7% of acceptable quality (e-Tables 2, 3). The reasons for excluded trials were irregular breathing patterns, signs of gas mixture changes, instable washin without peak in the expiration curve, instable washout, or swallowing.

Children with successful DTG-SBW tests were slightly older (1.1 years), had slightly lower Swiss-SEP (1.0 point), reported wheeze more frequently (5.3%) than the children with unsuccessful tests. All other anthropometric and questionnaire data were comparable (e-Table 4). Children with acceptable DTG-SBW tests had 72.3% A trials, 20.8% B trials, and 7.0% F trials, while this was 13.5%, 9.6%, and 77.0 % for the children with rejected data (e-Table 2, e-Figure 2). Age was associated with a higher success rate and test quality (e-Table 5). We explored differences in characteristics of study participants and tidal breathing during DTG-SBW between groups of quality control categories (e-Table 6). We did not observe trends deemed relevant for the interpretation of the variation in SIII_He-SF6_. Frequency of higher trial quality categories was associated with the number of acceptable trials (e-Table 7 and e-Figure 2, panel a) until a maximum of 4 trials (e-Table 8 and e-Figure 2, panel b). Indeed, we saw a slight learning effect, the later the trials the better the quality until 4 trials, after that again a decrease (e-Table 4, e-figure 2) perhaps because of exhaustion or loss of attention.

**DTG-SBW repeatability**

Two investigators performed independent quality control assessments on randomly selected 196 (3.8 %) out of 5223 DTG-SBW trials to assess inter-rater agreement. The inter-rater agreement was estimated as the percentage of agreement in quality control (acceptable vs. rejected test) and Cohen`s Kappa. The percentage of agreement between the two raters was 91% and Cohen`s Kappa was 0.64.

The median intra-test intra-test coefficient of variation (CV) [interquartile range, IQR] of SIII_He-SF6_ was 39.8 [22.0-70.9]%. We assessed the association between the log transformed CV and the magnitude of SIII_He-SF6,_ age, sex and variability in expired volume using scatterplots and Pearson’s correlation coefficients (e-Figure 3). The CV for SIII_He-SF6_ was not associated with the SIII_He-SF6_ value itself or sex, however it was associated with age and the tidal volume CV (e-Table 9).

Normalization of SIII_He-SF6_ using tidal volume is suggested to account for breathing pattern and reduce variability of the test[^1^](#_ENREF_1)^.^ Adjusting SIII_He-SF6_ for tidal volume slightly improved repeatability but introduced influences from auto-correlation and breathing pattern. The CV of SnIII_He-SF6_ was 31.6 [17.1-58.3]%. The log transformed CV of SnIII_He-SF6_ was associated with the SnIII_He-SF6_ value itself_,_ age and tidal volume variability, but not with sex (e-Table 9).

**DTG-SBW association with common predictors of lung function**

SnIII_He-SF6_ was associated with sex, age, height, BMI z-score in univariable and multivariable regression models (e-Table 11). In a multivariable regression model sex, age, and BMI z-score remained independent predictors of SnIII_He-SF6._ The SnIII_SF6-He_ decreased on average by 0.021 g.mol^-1^ per change from female to male and 0.007 g.mol^-1^ per one year increase in age, while it increased by 0.022 g.mol^-1^ per one z-score increase in BMI. SnIII_He-SF6_ was weakly associated with wheeze in unadjusted and adjusted multivariable linear regression models accounting for sex, age, height and BMI z-score (e-Table 11). The SnIII_He-SF6_ increased by 0.015 g.mol^-1^ in children with wheeze as compared with children without wheeze after adjusting for sex, age and BMI z-score. SnIII_He-SF6_ was associated with FEV_1,_ but not with FeNO or FEV_1_/FVC (e-Table 11).

We performed a *post hoc* analysis in the sub-group of children with extreme SIII_He-SF6_ values, i.e. median SIII_He-SF6_ ± 2 median absolute deviations (MAD). As expected from regression analysis, children with high SIII_He-SF6_ values (n = 178) had higher BMI, mean (SD) 0.74 (1.27) z-score, and prevalence of wheeze, 27.0 (44.5)%, as compared to children with low SIII_He-SF6_ values (n = 183) in whom BMI was lower, -0.38 (1.06) z-score, and prevalence of wheeze, 15.8 (36.5)%, was lower.

We acknowledge that the additional analyses require cautious interpretation due to multiple statistical testing. However, these data generally support the findings from the regression analyses applied to the whole cohort.

**References**

1. Singer F, Abbas C, Yammine S, Casaulta C, Frey U, Latzin P. Abnormal small airways function in children with mild asthma. *Chest.* 2014;145(3):492-499.

**

**

**e-Figure 1** Frequency of DTG-SBW measurements (N) *vs* timing of measurements (months, panel a). Scatterplot of the SIII_He-SF6_ values *vs* timing of measurements (collated per month, panel b). The closed circles display SIII_He-SF6_ values of children. We have excluded one outlier (SIII_He-SF6_ = 2.9 g.mol.L-1) in panel (b) to ease visualization. DTG-SBW: double-tracer gas (helium sulfur-hexafluoride) single-breath washout. SIII_He-SF6_: DTG-SBW derived phase III slope.


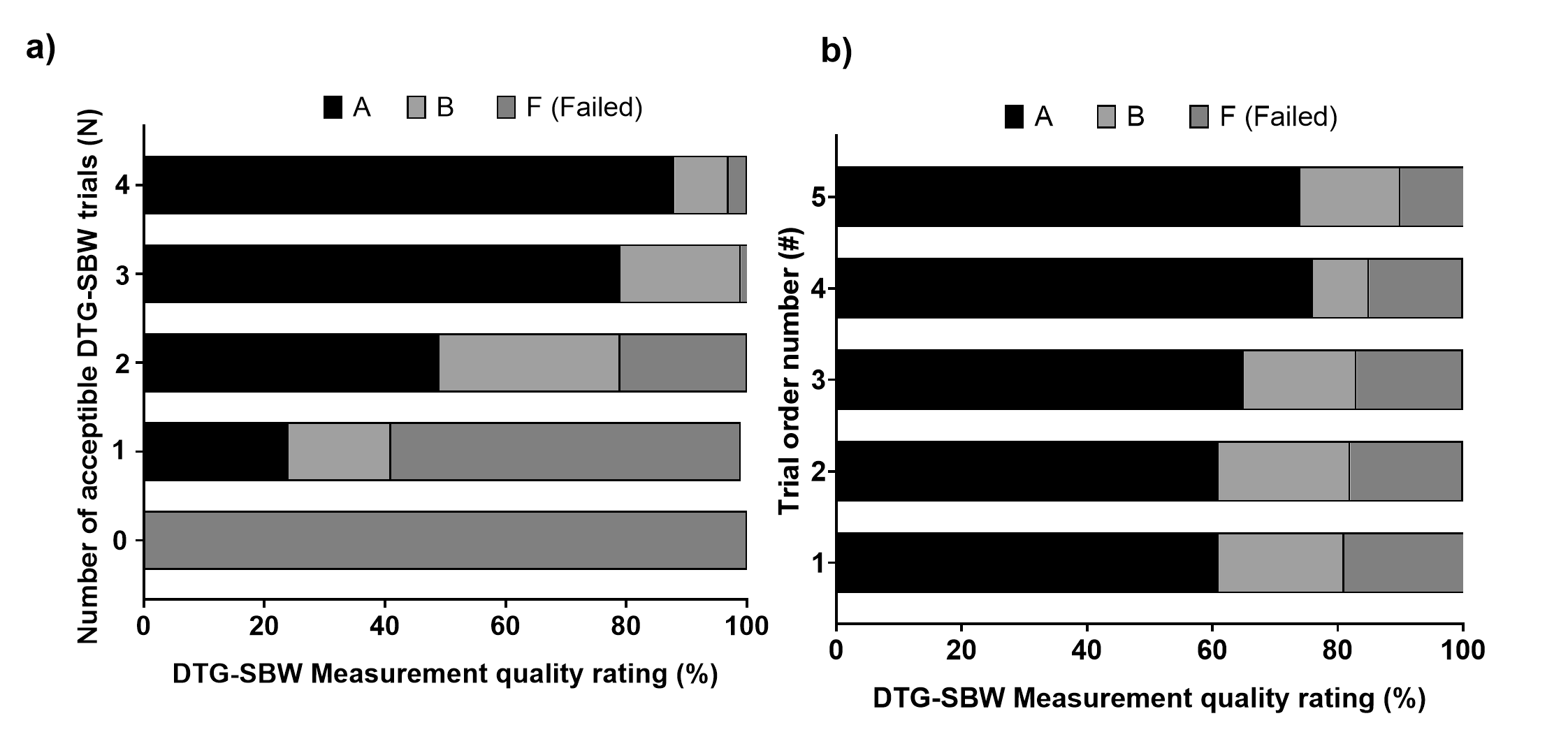


**e-Figure 2** Percentage component bar chart of the DTG-SBW measurement quality rating (%) *vs* frequency of acceptable DTG-SBW trials (N, panel a), and *vs* the trial order number (#, panel b) in 1782 children. The frequency of acceptable DTG-SBW trials summarizes the *number* of acceptable trials (0-4) per child per test occasion. The trial index depicts the *order* number (1-5) of a trial with a test occasion. The black and grey shades of colour display the DTG-SBW quality ratings (%). The black, light grey and dark grey bars display the quality rating categories A, B and failed (F), respectively. DTG-SBW: Double-tracer gas (helium sulfur-hexafluoride) single-breath washout.


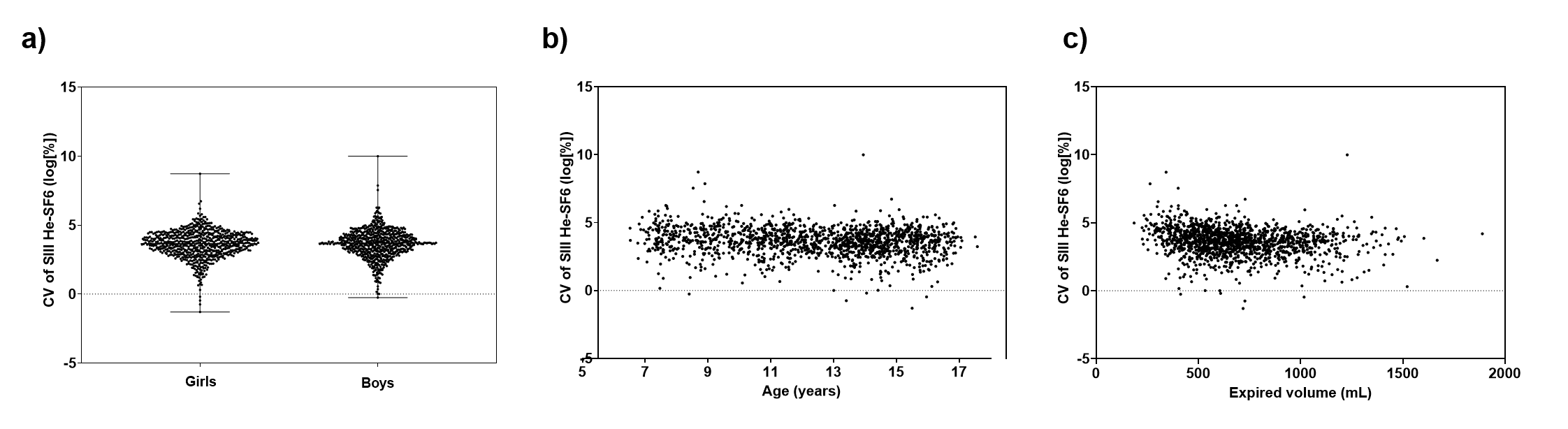


**e-Figure 3** Scatterplot of the log transformed coefficient of variation (CV) of DTG-SBW derived phase III slope (SIII_He-SF6_) *vs* sex (panel a), age in years (panel b), and the expired volume in mL (panel c) in 1449 children. CV: coefficient of variation. DTG-SBW: Double-tracer gas (helium sulfur-hexafluoride) single-breath washout. SIII_He-SF6_: DTG-SBW derived phase III slope.

| **e-Table 1.** **Double-tracer gas single-breath washout quality control.** | | | |
| --- | --- | --- | --- |
| **Criteria** | **A** | **B** | **Failed (F)** |
| Expired volume outliers | absent | absent | Expired volume per kilogram bodyweight < 5 mL/kg or > 25 mL/kg, or expired volume ≦ 0 mL. |
| Invalid SIII_co2_ | absent | absent | SIII_co2_ ≦ -1 g/mol |
| Signs of unregular tidal breathing | absent | unregular tidal breathing not affecting the washin or washout phase (such as by valve opening) | unregular tidal breathing during the washin, washout, or entire measurement |
| DTG molar mass offset | absent | DTG molar mass offset < 1.0 g/mol | DTG molar mass offset ≧ 1.0 g/mol |
| Signs of an unstable washin phase | absent | absent | unstable washin phase and a DTG mixture peak from the expiration curve misses |
| Signs of an unstable washout phase | absent | absent | unstable washout phase |
| Quality control protocol for DTG-SBW. Quality control category ratings include quality control categories A, B and Failed (F). Trials were only quality controlled if there were i. no evidence of air leaks as monitored by volume and molar mass (MM) signals, ii. similar flow-volume-loops in pre-test and test breaths, iii. breath volumes of the five tidal pre-test and the test breaths were within 10%, iv. inspiratory peak flow was below the range of the by-pass flow, v. if molar mass and CO_2_ phase III over at least 30% of expired volume, phase III was linear and present. DTG-SBW: double-tracer gas (helium sulfur-hexafluoride) single-breath washout. SIII_co2:_ carbon dioxide slope of phase III. | | | |

**e-Table 2** Characteristics of children invited and not invited for DTG-SBW

| **Participants** | **Invited for DTG-SBW** | **Not invited for DTG-SBW** |
| --- | --- | --- |
| Subjects, n | 1782 | 2102 |
| **General characteristics** |  |  |
| Males [%] | 889 [49.9] | 1056 [50.2] |
| Age (years) | 11.7 (2.8) | 12.4 (2.6) |
| BMI (z-score) | 0.1 (1.1) | 0.0 (1.2) |
| White Ethnicity [%] | 1349 [75.7] | 1591 [75.7] |
| Swiss-SEP (IQR) | 69.5 (62.1-75.9) | 70.8 (62.2-77.5) |
| Wheeze, n [%] | 322 [18.1] | 413 [19.7] |
| Hay fever, n [%] | 326 [18.3] | 441 [21.0] |
| Atopic dermatitis, n [%] | 188 [10.6] | 213 [10.1] |
| Asthma diagnosis, n [%] | 135 [7.6] | 158 [7.5] |
| Asthma medication, n [%] | 262 [14.7] | 315 [15.0] |
| **Lung function** |  |  |
| FeNO (ppb), median (IQR) | 11.0 (6.3-19.6) | 13.6 (8.0-23.3) |
| FEV_1_ (z-score) | -0.52 (0.97) | -0.56 (1.00) |
| FEV_1_/FVC (z-score) | -0.25 (1.04) | -0.17 (1.09) |
| SIII_He-SF6_ (g.mol.L^-1^) | -0.30 (0.54) |  |
| Data are presented as mean (SD) or percentage [%], unless indicated otherwise. All questionnaire data were parent reported. Asthma medication included any inhaled corticosteroids or short-acting or long-acting beta-agonists or systemic treatment such as leukotriene receptor antagonists. DTG-SBW: double-tracer gas (helium sulfur-hexafluoride) single-breath washout, BMI: body mass index. Swiss SEP: socioeconomic position in Switzerland. FeNO: fraction of exhaled nitric oxide, FEV_1_: forced expired volume in the first second, FVC: forced vital capacity, SIII_He-SF6:_ DTG-SBW slope of phase III. SIII_He-SF6_: DTG-SBW derived phase III slope. | | |

**e-Table 3** Success rates and quality control.

| **Participants** | **Invited for DTG-SBW** | **With acceptable DTG-SBW tests** | **With rejected DTG-SBW tests** |  |
| --- | --- | --- | --- | --- |
| Subjects | 1782 | 1449 | 333 |  |
| DTG-SBW | 1449 [81.3] | 1449 [100.0] | 0 [ 0.0] |  |
| FeNO | 1585 [89.0] | 1298 [89.6] | 287 [86.2] |  |
| FEV1 | 1448 [81.3] | 1193 [82.3] | 255 [76.6] |  |
| FEV1/FVC | 1252 [70.3] | 1029 [71.0] | 223 [67.0] |  |
| **Quality control** |  |  |  |  |
| Total trials | 5223 | 4398 | 825 |  |
| A-trials | 3288 [63.0] | 3177 [72.3] | 111 [13.5] |  |
| B-trials | 992 [19.0] | 913 [20.8] | 79 [9.6] |  |
| F-trials | 943 [18.0] | 308 [7.0] | 635 [77.0] |  |
| Data are presented as numbers and column percentages [%]. Quality control category ratings include quality control category A, B and Failed (F). Category A trials are considered of good quality, category B trials of moderate quality and were accepted. Category F trials were of inacceptable quality and therefore rejected. DTG-SBW: Double-tracer gas (helium sulfur-hexafluoride) single-breath washout. FeNO: Fraction of exhaled nitric oxide, FEV_1_: Forced expired volume in the first second, FVC: Forced vital capacity, SIII_He-SF6:_ DTG-SBW derived slope of phase III. | | | | |

**e-Table 4** Characteristics of children with acceptable vs rejected DTG-SBW trials

| **Participants** | **Invited for DTG-SBW** | **With acceptable DTG-SBW data** | **With rejected DTG-SBW data** |  |
| --- | --- | --- | --- | --- |
| Subjects, n | 1782 | 1449 | 333 |  |
| **General characteristics** |  |  |  |  |
| White Ethnicity [%] | 1349 [75.7] | 1107 [76.4] | 242 [72.7] |  |
| Swiss-SEP (IQR) | 69.5 (62.1-75.9) | 69.4 (62.1-75.0) | 70.9 (61.7-78.1) |  |
| Males [%] | 889 [49.9] | 719 [49.6] | 170 [51.1] |  |
| Age (years) | 11.7 (2.8) | 11.9 (2.7) | 10.8 (3.1) |  |
| BMI (z-score) | 0.1 (1.1) | 0.1 (1.1) | 0.0 (1.1) |  |
| Wheeze, n [%] | 322 [18.1] | 276 [19.1] | 46 [13.8] |  |
| Hay fever, n [%] | 326 [18.3] | 277 [19.1] | 49 [14.7] |  |
| Atopic dermatitis, n [%] | 188 [10.6] | 160 [11.0] | 28 [8.4] |  |
| Asthma diagnosis, n [%] | 135 [7.6] | 115 [7.9] | 20 [6.0] |  |
| Asthma medication, n [%] | 262 [14.7] | 218 [15.0] | 44 [13.2] |  |
| **Lung function** |  |  |  |  |
| FeNO (ppb), median (IQR) | 11.0 (6.3-19.6) | 11.1 (6.1-19.7) | 10.8 (7.2–19.5) |  |
| FEV_1_ (z-score) | -0.52 (0.97) | -0.54 (0.97) | -0.41 (0.98) |  |
| FEV_1_/FVC (z-score) | -0.25 (1.04) | -0.24 (1.06) | -0.27 (0.95) |  |
| SIII_He-SF6_ (g.mol.L^-1^) | -0.30 (0.54) | -0.30 (0.42) | -0.28 (0.90) |  |
| Data are presented as mean (SD) or percentage [%], unless indicated otherwise. All questionnaire data were parent reported. Asthma medication included any inhaled corticosteroids or short-acting or long-acting beta-agonists or systemic treatment such as leukotriene receptor antagonists. DTG-SBW: Double-tracer gas (helium sulfur-hexafluoride) single-breath washout, BMI: Body mass index. Swiss SEP: socioeconomic position index in Switzerland. FeNO: Fraction of exhaled nitric oxide, FEV_1_: Forced expired volume in the first second, FVC: Forced vital capacity, SIII_He-SF6:_ DTG-SBW derived slope of phase III. | | | | |

**e-Table 5** Association between success rate and quality control rating with potential predictors

| **Predictors** | **Correlation Coefficients** | **P-value** |  |
| --- | --- | --- | --- |
| *Success rate* |  |  |  |
| Age (year) | 0.20 | <0.001 |  |
| Sex (male vs female) | -0.01 | 0.656 |  |
| BMI (z-score) | 0.06 | 0.007 |  |
| Ethnicity (white vs non-white) | 0.03 | 0.211 |  |
| Wheeze vs no wheeze | 0.05 | 0.070 |  |
| *Quality control category* |  |  |  |
| Age (year) | 0.09 | <0.001 |  |
| Sex (male vs female) | -0.01 | 0.294 |  |
| BMI (z-score) | 0.07 | <0.001 |  |
| Ethnicity (white vs non-white) | 0.00 | 0.873 |  |
| Wheeze vs no wheeze | 0.02 | 0.115 |  |
| Associations between DTG-SBW success rate, quality control category and age, sex, BMI, ethnicity and wheeze. The success rate of DTG-SBW was calculated as the number of successful DTG-SBW trials as percentage of all DTG-SBW trials performed per subject. Quality control category ratings include quality control category A, B and Failed (F). Category A trials are considered of good quality, category B trials of moderate quality and were accepted. Category F trials were of inacceptable quality and therefore rejected. DTG-SBW trials from the category F are rejected and excluded from further analyses. Associations were assessed using Pearson correlation. BMI: body mass index. DTG-SBW: double-tracer (helium sulfur-hexafluoride) gas single-breath washout. | | | |

| **Quality control category** | **A** | **B** | **F** | **AB** | **ABF** |
| --- | --- | --- | --- | --- | --- |
| Subjects, n [%] | 843 [47.3] | 207 [11.6] | 143 [8.0] | 142 [8.0] | 35 [2.0] |
| **Characteristics** |  |  |  |  |  |
| Males [%] | 414 [49.1] | 108 [52.2] | 74 [51.8] | 68 [47.9] | 14 [40.0] |
| Age (years) | 11.9 (2.6) | 12.9 (2.5) | 10.9 (3.0) | 12.8 (2.6) | 11.3 (2.6) |
| BMI (z-score) | 0.2 (1.2) | 0.0 (1.1) | 0.1 (1.2) | 0.2 (1.2) | -0.2 (1.1) |
| White Ethnicity [%] | 645 [76.6] | 159 [76.8] | 100 [69.9] | 106 [74.7] | 28 [80.0] |
| Swiss-SEP | 69.7 (64.1- 74.8) | 68.4 (58.3-75.2) | 71.8 (62.9-77.8) | 69.5 (58.9-74.4) | 63.9 (56.7-69.5) |
| Wheeze, n [%] | 161 [19.1] | 39 [18.8] | 19 [13.3] | 25 [17.6] | 6 [17.1] |
| Hay fever, n [%] | 173 [20.5] | 33 [15.9] | 18 [12.6] | 25 [17.6] | 6 [17.1] |
| Atopic dermatitis, n [%] | 101 [12.0] | 16 [7.7] | 11 [7.7] | 17 [12.0] | 5 [14.3] |
| Asthma diagnosis, n [%] | 71 [8.4] | 15 [7.3] | 10 [7.0] | 13 [9.2] | 4 [11.4] |
| Asthma medication, n [%] | 136 [16.1] | 22 [10.6] | 18 [12.6] | 19 [13.4] | 3 [8.6] |
| **Lung function** |  |  |  |  |  |
| FeNO (ppb), median (IQR) | 9.9 (5.0-17.6) | 11.4 (8.1-21.3) | 12.2 (7.3-20.9) | 14.2 (9.1-25.3) | 14.3 (8.8-24.0) |
| FEV_1_ (z-score) | -0.5 (0.9) | -0.7 (1.0) | -0.3 (0.9) | -0.6 (1.0) | -0.8 (0.9) |
| FEV_1_/FVC (z-score) | -0.3 (1.0) | -0.3 (1.1) | -0.3 (0.9) | -0.2 (1.1) | -0.7 (1.0) |
| SIII_He-SF6_ (g.mol.L^-1^) | -0.3 (0.3) | -0.3 (0.2) | -0.2 (0.6) | -0.3 (0.6) | -0.5 (1.2) |
| **Variability** |  |  |  |  |  |
| CV SIII_He-SF6_ (%), median (IQR) | 43.3 (25.6-76.6) | 26.2 (14.6-44.2) | 49.0 (25.5-105.5) | 41.2 (23.4-63.3) | 70.2 (44.0-118.4) |
| CV expired volume (%), median (IQR) | 34.7 (20.3-66.5) | 20.8 (11.3-32.4) | 43.2 (18.9-89.1) | 35.9 (15.9-49.2) | 55.8 (32.7-91.8) |
| Data are presented as mean (SD) or percentage [%], unless indicated otherwise. All questionnaire data were parent reported. Quality control category ratings include quality control category A, B and Failed (F). Category A trials are considered of good quality, category B trials of moderate quality and were accepted. Category F trials were of inacceptable quality and therefore rejected. Asthma medication included any inhaled corticosteroids or short-acting or long-acting beta-agonists or systemic treatment such as leukotriene receptor antagonists. DTG-SBW: Double-tracer gas (helium sulfur-hexafluoride) single-breath washout, BMI: Body mass index. CV: coefficient of variation and displayed as median [interquartile range]. Swiss SEP: socioeconomic position index in Switzerland. FeNO: Fraction of exhaled nitric oxide, FEV_1_: Forced expired volume in the first second, FVC: Forced vital capacity, SIII_He-SF6:_ DTG-SBW derived slope of phase III. | | | | | |

**e-Table 6** Characteristics of DTG-SBW quality control groups

**e-Table 7** DTG-SBW measurement quality rating by frequency of acceptable DTG-SBW trials

| Number of acceptable DTG-SBW trials (N) | DTG-SBW Measurement quality rating | N [%] |  |
| --- | --- | --- | --- |
| >3 | A | 556 [88.0] |  |
|  | B | 55 [8.7] |  |
|  | F | 21 [3.3] |  |
|  | ABF | 632 [100.0] |  |
| 3 | A | 2053 [78.7] |  |
|  | B | 512 [19.6] |  |
|  | F | 43 [1.7] |  |
|  | ABF | 2608 [100.0] |  |
| 2 | A | 568 [49.1] |  |
|  | B | 346 [29.9] |  |
|  | F | 244 [21.1] |  |
|  | ABF | 1158 [100.0] |  |
| 1 | A | 111 [24.3] |  |
|  | B | 79 [17.3] |  |
|  | F | 267 [58.4] |  |
|  | ABF | 457 [100.0] |  |
| 0 | A | 0 [0.0] |  |
|  | B | 0 [0.0] |  |
|  | F | 368 [100.0] |  |
|  | ABF | 368 [100.0] |  |
| DTG-SBW measurement quality rating vs frequency of acceptable DTG-SBW trials. In1782 DTG-SBW test occasions 5223 trials were obtained. We stratified these trials according to the number of acceptable trials per children (from 0 to >3) per test occasion. For these groups, we assessed the numbers [percentage] of trials including the distribution of quality control categories. Category A trials are considered of good quality, category B trials of moderate quality and were accepted. Category F trials were of inacceptable quality and therefore rejected. DTG-SBW: Double-tracer gas (helium sulfur-hexafluoride) single-breath washout, N: number of DTG-SBW trials. | | | |

**e-Table 8** DTG-SBW measurement quality rating by DTG-SBW trial order number

| Trial order number (#) | DTG-SBW Measurement quality rating | N [%] |  |
| --- | --- | --- | --- |
| 1 | A | 1073 [60.9] |  |
|  | B | 344 [19.5] |  |
|  | F | 345 [19.6] |  |
|  | ABF | 1762 [100.0] |  |
| 2 | A | 1060 [61.0] |  |
|  | B | 363 [20.9] |  |
|  | F | 315 [18.1] |  |
|  | ABF | 1738 [100.0] |  |
| 3 | A | 912 [65.1] |  |
|  | B | 249 [17.8] |  |
|  | F | 240 [17.1] |  |
|  | ABF | 1401 [100.0] |  |
| 4 | A | 168 [76.4] |  |
|  | B | 20 [9.1] |  |
|  | F | 32 [14.6] |  |
|  | ABF | 220 [100.0] |  |
| >=5 | A | 75 [73.5] |  |
|  | B | 16 [15.7] |  |
|  | F | 11 [10.8] |  |
|  | ABF | 102 [100.0] |  |
| DTG-SBW measurement quality rating vs DTG-SBW trial order number. In1782 DTG-SBW test occasions 5223 trials were obtained. We stratified these trials according to their trial order number (i.e. index) (from 0 to >5) per test occasion. For these groups, we assessed the numbers [percentage] of trials including the distribution of quality control categories. Category A trials are considered of good quality, category B trials of moderate quality and were accepted. Category F trials were of inacceptable quality and therefore rejected. DTG-SBW: Double-tracer gas (helium sulfur-hexafluoride) single-breath washout, #: trial order number. N: number of DTG-SBW trials. | | | |

**e-Table 9** Association between variability of DTG-SBW and potential explanatory variables

|  | **Correlation Coefficient** | **P-value** |  |
| --- | --- | --- | --- |
| *Coefficient of variation of SIII_He-SF6,_ log[%]* |  |  |  |
| SIII_He-SF6_ (g.mol.L^-1^) | -0.023 | 0.373 |  |
| Age (years) | -0.147 | <0.001 |  |
| Male sex | -0.021 | 0.432 |  |
| CV_VT_ (%) | 0.388 | <0.001 |  |
| *Coefficient of variation, SnIII_He-SF6 ,_ log[%]* |  |  |  |
| SnIII_He-SF6_ (g.mol^-1^) | 0.223 | <0.001 |  |
| Age (years) | -0.141 | <0.001 |  |
| Male sex | -0.015 | 0.572 |  |
| CV_VT_ (%) | 0.279 | <0.001 |  |
| Associations between log transformed intra-test coefficient of variation of phase III slope derived from DTG-SBW outcomes and phase III slope values, age, sex and intra-test coefficient of variation of the expired tidal volume per kilogram. Associations were assessed using Pearson correlation. DTG-SBW: double-tracer (helium sulfur-hexafluoride) gas single-breath washout, SIII_He-SF6_: DTG-SBW derived slope of phase III, SnIII_He-SF6_: DTG-SBW derived slope of phase III normalized for expired tidal volume, CV: coefficient of variation, VT: expired tidal volume. | | | |

**e-Table 10** Characteristics of children with acceptable DTG-SBW and with or without wheeze

|  | **Children with wheeze** | **Children without wheeze** | **All children** | **P-value** |
| --- | --- | --- | --- | --- |
| Study participants, n | 276 | 1025 | 1449 |  |
| **General characteristics** |  |  |  |  |
| Males [%] | 125 [45.3] | 510 [49.8] | 719 [49.6] | 0.188 |
| Age (years) | 12.3 (2.6) | 11.6 (2.6) | 11.9 (2.7) | <0.001 |
| BMI (z-score) | 0.3 (1.2) | 0.0 (1.1) | 0.1 (1.1) | 0.002 |
| White Ethnicity [%] | 206 [74.6] | 784 [76.5.8] | 1107 [76.4] | 0.143 |
| Swiss-SEP (IQR) | 69.3 (60.7-73.9) | 69.5 (62.3-75.5) | 69.4 (62.1-75.0) | 0.340 |
| Wheeze, n [%] | 276 [100.0] | 0 [0.0] | 276 [19.1] |  |
| Hay fever, n [%] | 113 [40.9] | 162 [15.8] | 277 [19.1] | <0.001 |
| Atopic dermatitis, n [%] | 42 [15.2] | 117 [11.4] | 160 [11.0] | 0.076 |
| Asthma diagnosis, n [%] | 70 [25.4] | 44 [4.3] | 115 [7.9] | <0.001 |
| Asthma medication, n [%] | 119 [43.1] | 96 [9.4] | 218 [15.0] | <0.001 |
| **Lung function** |  |  |  |  |
| FeNO (ppb), median (IQR) | 14.2 (7.2–28.8) | 9.9 (5.5–17.3) | 10.7 (5.8 to 18.8) | <0.001 |
| FEV_1_ (z-score) | -0.69 (1.00) | -0.48 (0.95) | -0.53 (0.97) | 0.004 |
| FEV_1_/FVC (z-score) | -0.52 (1.10) | -0.16 (1.04) | -0.24 (1.07) | <0.001 |
| SIII_He-SF6_ (g.mol.L^-1^) | -0.26 (0.27) | -0.33 (0.38) | -0.32 (0.36) | 0.003 |
| Data are presented as mean (SD) or percentage [%], unless indicated otherwise, and compared between children with vs without wheeze using unpaired t-tests or Mann-Whitney tests as appropriate. All questionnaire data were parent reported. Asthma medication included any inhaled corticosteroids or short-acting or long-acting beta-agonists or systemic treatment such as leukotriene receptor antagonists, DTG-SBW: Double-tracer gas (helium sulfur-hexafluoride) single-breath washout, BMI: Body mass index, Swiss SEP: socioeconomic position index in Switzerland. FeNO: Fraction of exhaled nitric oxide, FEV_1_: Forced expired volume in the first second, FVC: forced vital capacity, SIII_He-SF6:_ DTG-SBW derived slope of phase III. | | | | |

**e-Table 11** Non-adjusted and adjusted association between SnIII_He-SF6_ and potential predictors

| **Predictors** | **Regression coefficients** | **95% CI** | **P-value** |  |
| --- | --- | --- | --- | --- |
| **Anthropometrics** |  |  |  |  |
| Sex males vs females | -0.019 | -0.032 to -0.006 | 0.004 |  |
| Age (year) | -0.005 | -0.008 to -0.003 | <0.001 |  |
| Height (cm) | 0.000 | -0.001 to 0.000 | 0.007 |  |
| Weight (kg) | 0.000 | 0.000 to 0.001 | 0.135 |  |
| BMI (z-score) | 0.020 | 0.014 to 0.026 | <0.001 |  |
| **Symptoms** |  |  |  |  |
| wheeze vs no wheeze | 0.016 | 0.000 to 0.032 | 0.052 |  |
| wheeze vs no wheeze, adjusted | 0.016 | 0.000 to 0.032 | 0.049 |  |
| **Lung function** |  |  |  |  |
| FeNO (quintiles ) | -0.004 | -0.009 to 0.001 | 0.122 |  |
| FEV1 (z-score) | 0.009 | 0.001 to 0.016 | 0.020 |  |
| FEV1/FVC (z-score) | 0.006 | 0.000 to 0.013 | 0.066 |  |
| Associations between SnIII_He-SF6_ and potential predictors were assessed using uni- and multivariable linear regression models. Predictors were age, sex, height, weight and BMI; wheeze, and FeNO, FEV_1_ and FEV_1_/FVC. Wheeze was included as a binary variable (i.e. yes or no) and FeNO as data-driven quintiles ensuring balanced observations per category, all other variables were included as continuous variables with their original scale. The quintile boundaries for FeNO were: 0.0-4.9, 5.0-8.8, 8.9-13.8, 13.9-23.4, and 23.5-197.0 ppb, respectively. A multivariable linear regression model was used to assess which anthropometric variables were independent predictors of SIII_He-SF6_, and the independent predictors sex, age, height and BMI z-score were used to adjust the association of SIII_He-SF6_ with wheeze. All associations described the change in SnIII_He-SF6_ in g.mol^-1^ induced by one unit increase in the potential predictor. CI: Confidence interval, DTG-SBW: Double-tracer gas (helium sulfur-hexafluoride) single-breath washout, SnIII_He-SF6_: DTG-SBW derived slope of phase III normalized for tidal volume, BMI: Body mass index (z-score). FeNO: Fraction of exhaled nitric oxide, FEV_1_: Forced expired volume in the first second, FVC: Forced vital capacity. | | | | |

| **e-Table 12** Non-adjusted and adjusted association between SIII_He-SF6_ and potential predictors in children with a BMI z-score>1 | | | |
| --- | --- | --- | --- |
| **Predictors** | **Regression coefficients** | **95% CI** | **P-value** |
| **Anthropometrics** | | | |
| Sex (male vs female) | 0.005 | -0.061 to 0.071 | 0.880 |
| Age (year) | 0.035 | 0.023 to 0.048 | <0.001 |
| Height (cm) | 0.006 | 0.004 to 0.008 | <0.001 |
| Weight (kg) | 0.005 | 0.003 to 0.007 | <0.001 |
| BMI (z-score) | 0.072 | 0.001 to 0.131 | 0.017 |
| **Symptoms** |  |  |  |
| wheeze vs no wheeze | 0.062 | 0.001 to 0.124 | 0.048 |
| wheeze vs no wheeze, adjusted | 0.039 | -0.021 to 0.099 | 0.193 |
| **Lung function** |  |  |  |
| FeNO (quintiles ) | 0.013 | -0.012 to 0.038 | 0.291 |
| FEV_1_ (z-score) | -0.005 | -0.042 to 0.032 | 0.784 |
| FEV_1_/FVC (z-score) | 0.013 | -0.026 to 0.052 | 0.512 |
| Associations between SIII_He-SF6_ and potential predictors were assessed using uni- and multivariable linear regression models in children with a BMI z-score >1. Predictors were age, sex, height, weight and BMI; wheeze, FeNO, FEV_1_ and FEV_1_/FVC. Wheeze was included as a binary variable (i.e. yes or no) and FeNO as data-driven quintiles ensuring balanced observations per category, all other variables were included as continuous variables with their original scale. The quintile boundaries for FeNO were: 0.0-4.9, 5.0-8.8, 8.9-13.8, 13.9-23.4, and 23.5-197.0 ppb, respectively. A multivariable linear regression model was used to assess which anthropometric variables were independent predictors of SIII_He-SF6_, and the independent predictors age and BMI z-score were used to adjust the association of SIII_He-SF6_ with wheeze. All associations described the change in SIII_He-SF6_ in g.mol.L^-1^ induced by one unit increase in the predictor. CI = confidence interval, DTG-SBW = double-tracer (helium sulfur-hexafluoride) gas single breath washout, SIII_He-SF6_: DTG-SBW derived slope of phase III, BMI: body mass index (z-score). FeNO: fraction of exhaled nitric oxide, FEV_1_: forced expired volume in the first second, FVC: forced vital capacity. | | | |
